# HDAC3 and HDAC8 are required for cilia assembly and elongation

**DOI:** 10.1242/bio.043828

**Published:** 2019-07-30

**Authors:** Seon-ah Park, Hyunjeong Yoo, Jae Hong Seol, Kunsoo Rhee

**Affiliations:** Department of Biological Sciences, Seoul National University, Seoul 08826, Korea

**Keywords:** Cilium, Ciliogenesis, Histone deacetylases, HDAC8

## Abstract

Cilia are extended from mother centrioles in quiescent G0/G1 cells and retracted in dividing cells. Diverse post-translational modifications play roles in the assembly and disassembly of the cilium. Here, we examined class I histone deacetylases (HDACs) as positive regulators of cilia assembly in serum-deprived RPE1 and HK2 cells. We observed that the number of cells with cilia was significantly reduced in HDAC3- and HDAC8-depleted cells. The ciliary length also decreased in HDAC3- and HDAC8-depleted cells compared to that in control cells. A knockdown-rescue experiment showed that wild-type HDAC3 and HDAC8 rescued the cilia assembly and ciliary length in HDAC3- and HDAC8-depleted cells, respectively; however, deacetylase-dead HDAC3 and HDAC8 mutants did not. This suggests that deacetylase activity is critical for both HDAC3 and HDAC8 function in cilia assembly and ciliary length control. This is the first study to report that HDACs are required for the assembly and elongation of the primary cilia.

## Abstract (precis)

**Summary:** We identified that HDAC3 and HDAC8 are required for the assembly and elongation of the primary cilia.

## INTRODUCTION

The primary cilium is a non-motile, sensory organelle that protrudes from the cell surface. It transduces specific intercellular signals to the cell body and regulates various cellular events for growth and differentiation. Defects in ciliary structure and function lead to pleotropic disorders called ciliopathies, which are characterized by cystic kidney disease, obesity, mental retardation and retinal degeneration (Goetz and Anderson, 2010).

The primary cilium originates from a mother centriole in a quiescent G0/G1 cell. The first visible sign of ciliogenesis may be the accumulation of Golgi-derived ciliary vesicles in the vicinity of distal appendages of mother centrioles (Sánchez and Dynlacht, 2016). Vesicular fusion produces a membranous cap on the distal tip of the mother centriole. The mother centriole then differentiates into a basal body and extends microtubules underneath the cap. Finally, the nascent cilium docks at the cytoplasmic membrane by fusion with the ciliary sheath, establishing the continuity of these compartments (Sung and Leroux, 2013). Disassembly of the primary cilium is triggered by the replenishment of serum growth factors. Tubulin deacetylation precedes regression of the ciliary axoneme (Li and Yang, 2015).

Diverse regulatory mechanisms control the assembly and disassembly of the primary cilium. Protein phosphorylation is an important regulatory mechanism, and a number of protein kinases have been determined to play essential roles at specific steps of cilia assembly and disassembly (Pugacheva et al., 2007; Goetz et al., 2012; Kuhns et al., 2013; Kim et al., 2015b; Miyamoto et al., 2015; Sanchez and Dynlacht, 2016). Protein acetylation/deacetylation is another post-translational modification that regulates the functions of the cilium (Li and Yang, 2015). Several protein acetyltransferases and deacetylases have been determined to play essential roles in ciliogenesis. α-Tubulin acetyltransferase 1 (αTAT1) is responsible for α-tubulin acetylation in ciliary microtubules and facilitates cilia assembly (Akella et al., 2010; Shida et al., 2010). In contrast, HDAC6 and SIRT2 induce cilia disassembly by deacetylating α-tubulin in the microtubule of the axoneme (Pugacheva et al., 2007; Zhou et al., 2014). HDAC2 is essential for suppression of cilia formation by positively regulating Aurora A levels in dividing tumor cells (Kobayashi et al., 2017). Although the importance of HDACs in cilia disassembly has been revealed, as of yet, no deacetylase has been identified as a positive regulator of cilia assembly.

The eleven known HDACs are grouped into classes I, II and IV based on their structural similarity (Table S1; Yang and Seto, 2008). It is generally known that class I HDACs are ubiquitously expressed and crucial for transcriptional repression and epigenetic landscaping (Yang and Seto, 2008). Class II HDACs regulate cytoplasmic processes or function as signal transducers that shuttle between the cytoplasm and the nucleus (Yang and Seto, 2008). However, recent experimental evidence suggests that class I HDACs also function in various biological events through their non-histone substrates. For example, HDAC8 is known to deacetylate cortactin to promote actin filament polymerization and smooth muscle contraction (Li et al., 2014). HDAC1 is localized at the centrosome and plays a role in centriole biogenesis (Sakai et al., 2002; Ling et al., 2012). HDAC3 is known to shuttle in and out of the nucleus, while other class I HDACs are primarily localized in the nucleus (Yang et al., 2002) and deacetylate myocyte enhancer factor 2, which regulates embryo development, including muscle development (Grégoire et al., 2007). Meanwhile, HDAC8 localizes at the spindle poles to function in spindle assembly and chromosome alignment during mouse oocyte meiosis (Zhang et al., 2017). These reports revealed that the biological functions of class I HDACs are not limited to epigenetic transcriptional regulation but are expanded to include diverse cellular processes.

A long-term goal of our study is to elucidate the functions of class I HDACs in cilium assembly and disassembly. In this work, we examined deacetylases that function as positive regulators of primary cilia formation in serum-deprived cells. Our results revealed that selected HDACs, such as HDAC3 and HDAC8, are required for the assembly and elongation of the primary cilia.

## RESULTS

### Identification of HDACs for cilia assembly and elongation

**Fig. 1.**
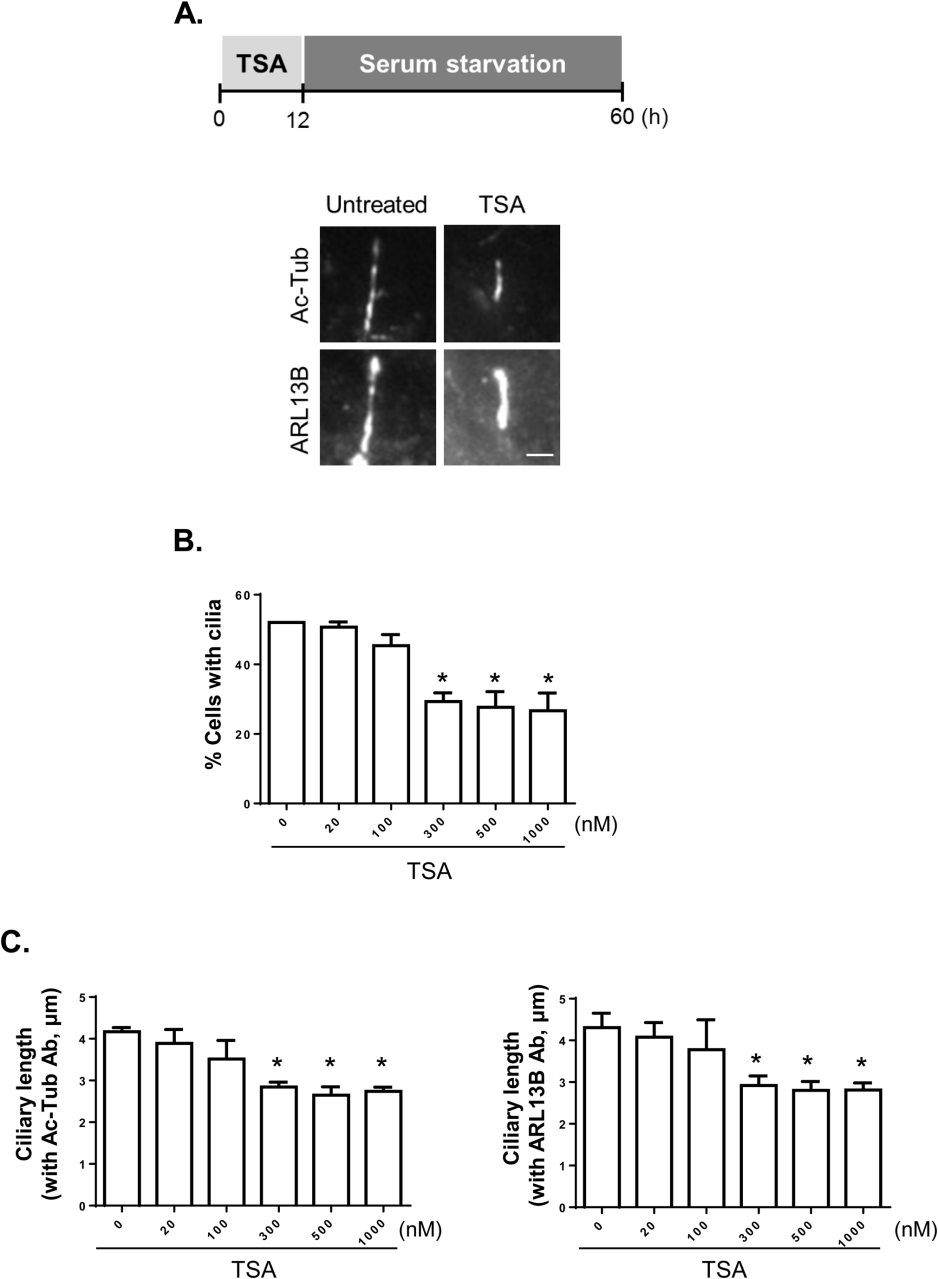
**Involvement of the class I HDACs in the cilia formation.** (A) RPE1 cells were pre-treated with TSA for 12 h and cultured in serum-deprived medium. Forty-eight hours later, the cells were immunostained with antibodies specific to acetylated α-tubulin and ARL13B. Scale bar: 2 μm. (B) The number of cells with cilia was determined. Greater than 100 cells per experimental group were counted in three independent experiments. (C) Ciliary length was measured. Greater than 30 cilia per experimental group were measured in three independent experiments. Values are means and s.e.m. Statistical significance was analyzed using one-way ANOVA and is indicated by *(*P*<0.05).

We began our study examining the effects of trichostatin A (TSA), a selective inhibitor of HDACs, on cilia formation. The metal binding domain of TSA coordinates a catalytic Zn(II) and occupies the active sites of HDACs (Furumai and Horinouchi, 2011). RPE1 cells, which are human retinal pigmented epithelial cells, were treated with TSA for 12 h and cultured in serum-deprived medium for cilia assembly (Fig. 1). Cilia were visualized with antibodies specific to acetylated α-tubulin and ARL13B (Fig. 1A). The number of ciliated cells was reduced in cells treated with 300 nM or higher concentrations of TSA compared to that in control cells (Fig. 1B). The ciliary length was also shortened following treatment with 300 nM or higher concentrations of TSA (Fig. 1C). These results suggest that class I HDACs are required for the assembly and elongation of the cilia because the enzymatic activities of class I HDACs were almost completely inhibited by 300 nM TSA.

**Fig. 2.**
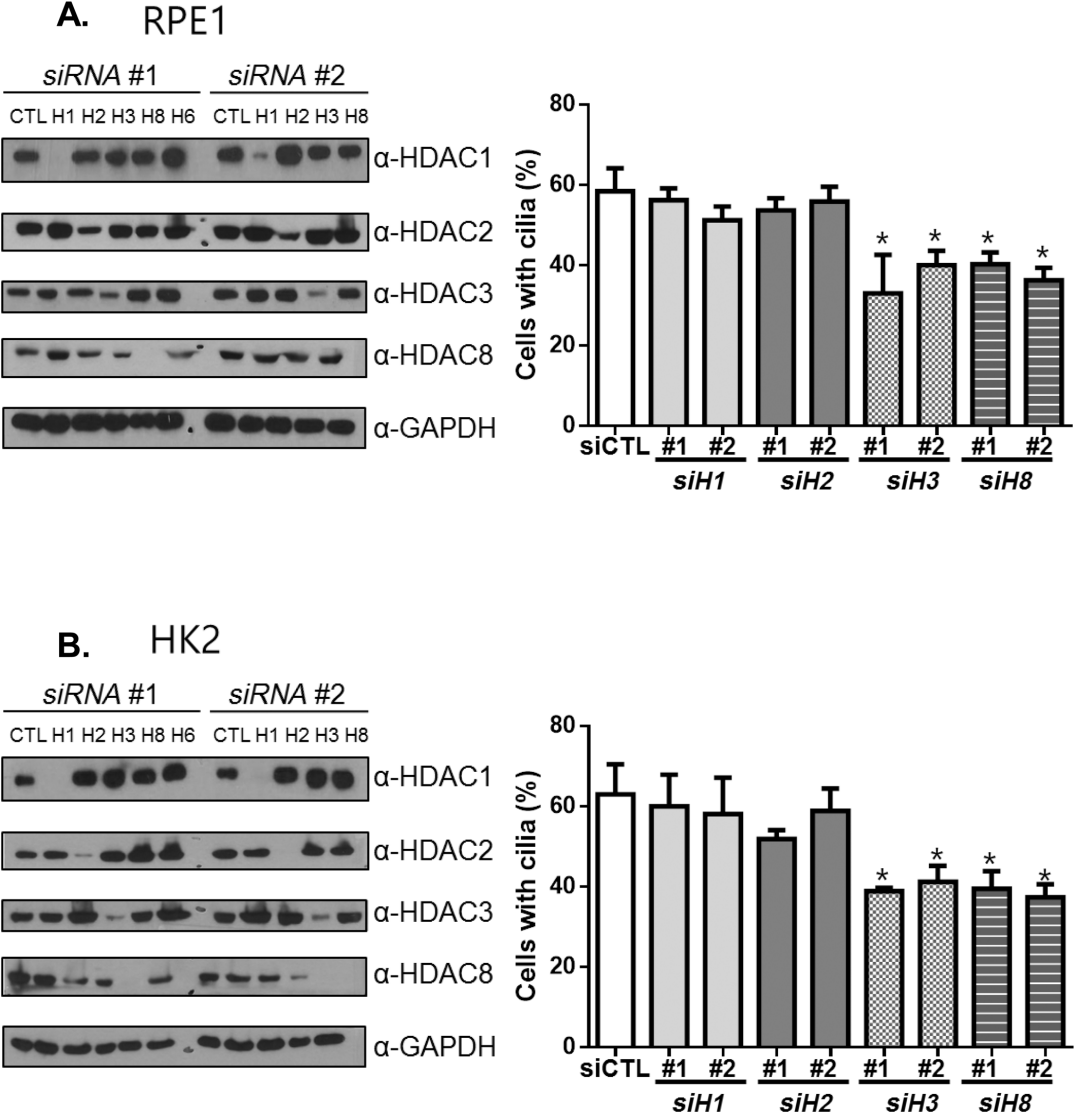
**Involvement of the selected HDACs in cilia formation.** The RPE1 (A) and HK2 (B) cells were transfected with two different sets of siRNAs specific to *HDAC1*, *2*, *3*, *8* or *6*. Forty-eight hours later, the cells were transferred to serum-deprived medium and cultured for additional 48 h. The cell lysates were subjected to immunoblot analysis to confirm depletion of HDACs. Intensities of the specific bands were measured and statistically analyzed in Fig. S1. The number of cells with cilia was counted and statistically analyzed. Greater than 100 cells per group were counted in three independent experiments. Values are means and s.e.m. Statistical significance was analyzed using one-way ANOVA and is indicated by *(*P*<0.05).

To identify which class I HDACs are required for cilium formation, we determined the number of cells with cilia after the depletion of class I HDACs (HDAC1, HDAC2, HDAC3 and HDAC8) and a class II HDAC (HDAC6) (Fig. 2A,B). We used two different cell lines: RPE1 (a retinal pigmented epithelial cell line) and HK2 (a human kidney tubular epithelial cell line). Both cell lines originate from tissues in which cilia play critical roles in the physiological functions of the cells (Seeger-Nukpezah et al., 2012). Two different kinds of siRNAs per HDAC species were used to reduce possible artefacts derived from the off-target effects of siRNAs. Immunoblot analyses confirmed the depletion of the target HDACs (Fig. 2A,B). In fact, siRNA transfection specifically depleted the corresponding HDACs with little effect on the cellular levels of other HDAC proteins (Fig. S1). However, we do not rule out a compensatory HDAC pathway which indirectly affects cilia assembly. We observed that approximately 70% and 60% of the control cells formed cilia in control RPE1 and HK2 cells, respectively (Fig. 2A,B). The depletion of HDAC3 and HDAC8 significantly reduced the number of cells with cilia in both the RPE1 and HK2 cells compared to those in the corresponding control cells (Fig. 2A,B). FACS revealed that over 90% of the control RPE1 cells were already arrested at G0/G1 phase (Fig. S2). Similar cell cycle patterns were also detected in both the HDAC3- and HDAC8-depleted RPE1 cells (Fig. S2). Therefore, we ruled out the possibility that the cilia assembly was indirectly affected by specific cell cycle arrest in the HDAC-depleted cells, at least in our experimental conditions. Taken together, these results suggest that HDAC3 and 8 might be involved in cilia assembly.

**Fig. 3.**
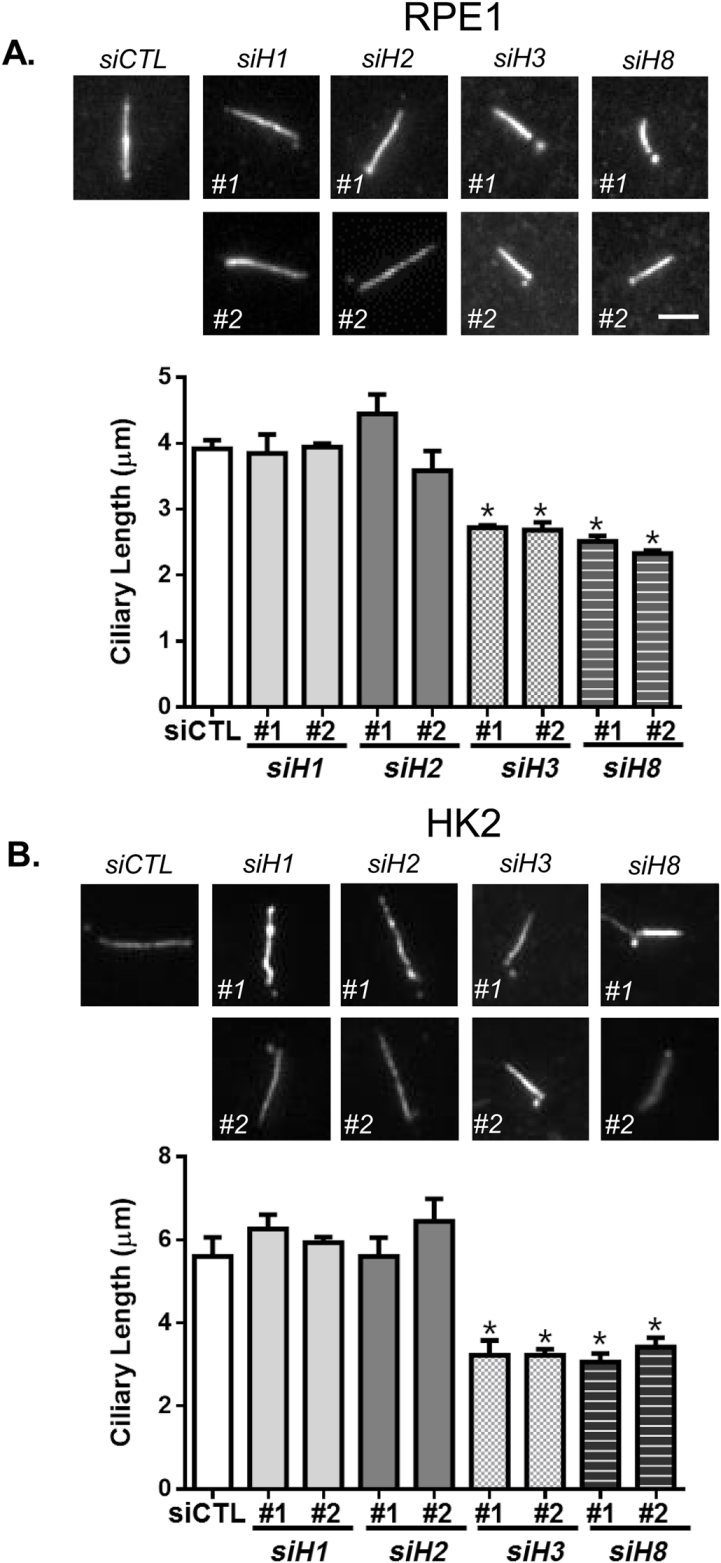
**Class I HDACs for the ciliary length control.** The RPE1 (A) and HK2 (B) cells were transfected with two sets of siRNAs specific to the class I *HDAC*s and *HDAC6*, and cultured in serum-deprived medium for 48 h. The cells were immunostained with the acetylated α-tubulin antibody. Scale bar: 2 μm. Ciliary length was measured. Greater than 30 cilia per experimental group were measured in three independent experiments. Values are means and s.e.m. Statistical significance was analyzed using one-way ANOVA and is indicated by * (*P*<0.05).

We determined the ciliary length in HDAC-depleted RPE1 cells (Fig. 3A). The depletion of HDAC3 and HDAC8 resulted in a reduction in ciliary lengths compared to those in control cells (Fig. 3A). It is interesting that the average ciliary length of HK2 cells was longer than that of RPE1 cells (Fig. 3B). The ciliary lengths of both HDAC3- and HDAC8-depleted HK2 cells were also shortened compared to those of the control HK2 cells (Fig. 3B). These results suggest that both HDAC3 and HDAC8 are involved in controlling cilia length.

### Both HDAC3 and HDAC8 are required for cilia assembly

We determined cilia assembly after knockdown for different time periods. RPE1 cells were transfected with *siHDAC8* for 6, 24 or 48 h and then cultured for 48 h in serum-deprived medium (Fig. S3). As expected, cellular HDAC8 levels were gradually reduced compared to those in control cells and eventually undetectable 48 h after siRNA transfection (Fig. S3). Cilia assembly was also reduced after knockdown in a time-dependent manner (Fig. S3). The ciliary lengths were sufficiently shortened 24 h after transfection (Fig. S3). These results suggest that the cilia assembly depends on cellular HDAC8 levels.

**Fig. 4.**
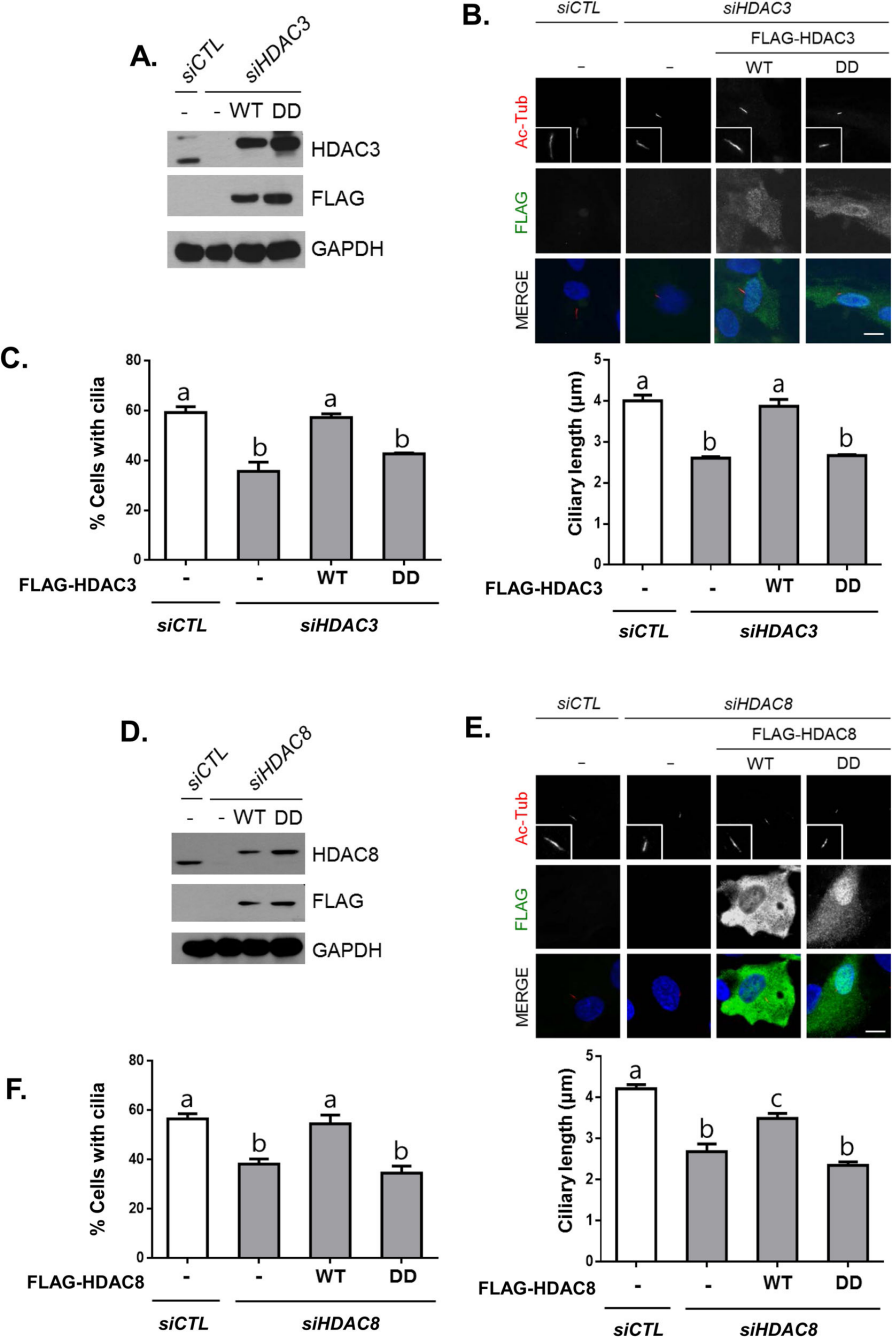
**The deacetylase activity of HDAC3 and HDAC8 is required for cilia formation and ciliary length control.** The siRNA-resistant forms of wild type (WT) and deacetylase-dead (DD) FLAG-HDAC3 (A–C) and HDAC8 (D–F) were stably transfected into RPE1 cells and depleted with transfection of specific siRNAs. (A,D) Depletion and ectopic expression of HDAC3 and HDAC8 were determined with immunoblot analyses. (B,E) After cultured in serum-deprived medium for 48 h, the cells were co-immunostained with antibodies specific to acetylated α-tubulin and FLAG. Scale bars: 10 μm. (C,F) The number of cells with cilia was determined. Greater than 100 cells per group were counted in three independent experiments. Ciliary length was measured. Greater than 30 cilia per experimental group were measured in five independent experiments. Statistical significance was analyzed using one-way ANOVA and is indicated by lower case letters (*P*<0.05).

We performed knockdown-rescue experiments to determine the importance of the deacetylase activity of HDAC3 in cilia formation. We generated stable RPE1 cell lines in which siRNA-resistant forms of wild-type and deacetylase-dead HDAC3 constructs were expressed. In the deacetylase-dead mutant, the arginine residue at the active site was substituted with proline residue (Watson et al., 2012). We then transfected *siHDAC3* into the stable lines to deplete endogenous HDAC3. Immunoblot analyses confirmed that comparable amounts of ectopic HDAC3 were expressed in HDAC3-depleted RPE1 cells (Fig. 4A,B). The cells were cultured in serum-deprived medium to induce cilia formation. Ectopic HDAC3 rescued the number of cells with cilia and ciliary length observed in the HDAC3-depleted cells (Fig. 4C). However, the deacetylase-dead HDAC3 mutant failed to rescue the cilia assembly and ciliary length observed in the HDAC3-depleted cells (Fig. 4C). We also performed identical knockdown-rescue experiments on HDAC8 and observed similar results (Fig. 4D–F). In the deacetylase-dead mutant of HDAC8, the histidine residues at the active site were substituted with alanine residues (Gantt et al., 2016; Joseph et al., 2000). These results indicate that the deacetylase activity of both HDAC3 and HDAC8 are essential for their function in cilia assembly and ciliary length control.

**Fig. 5.**
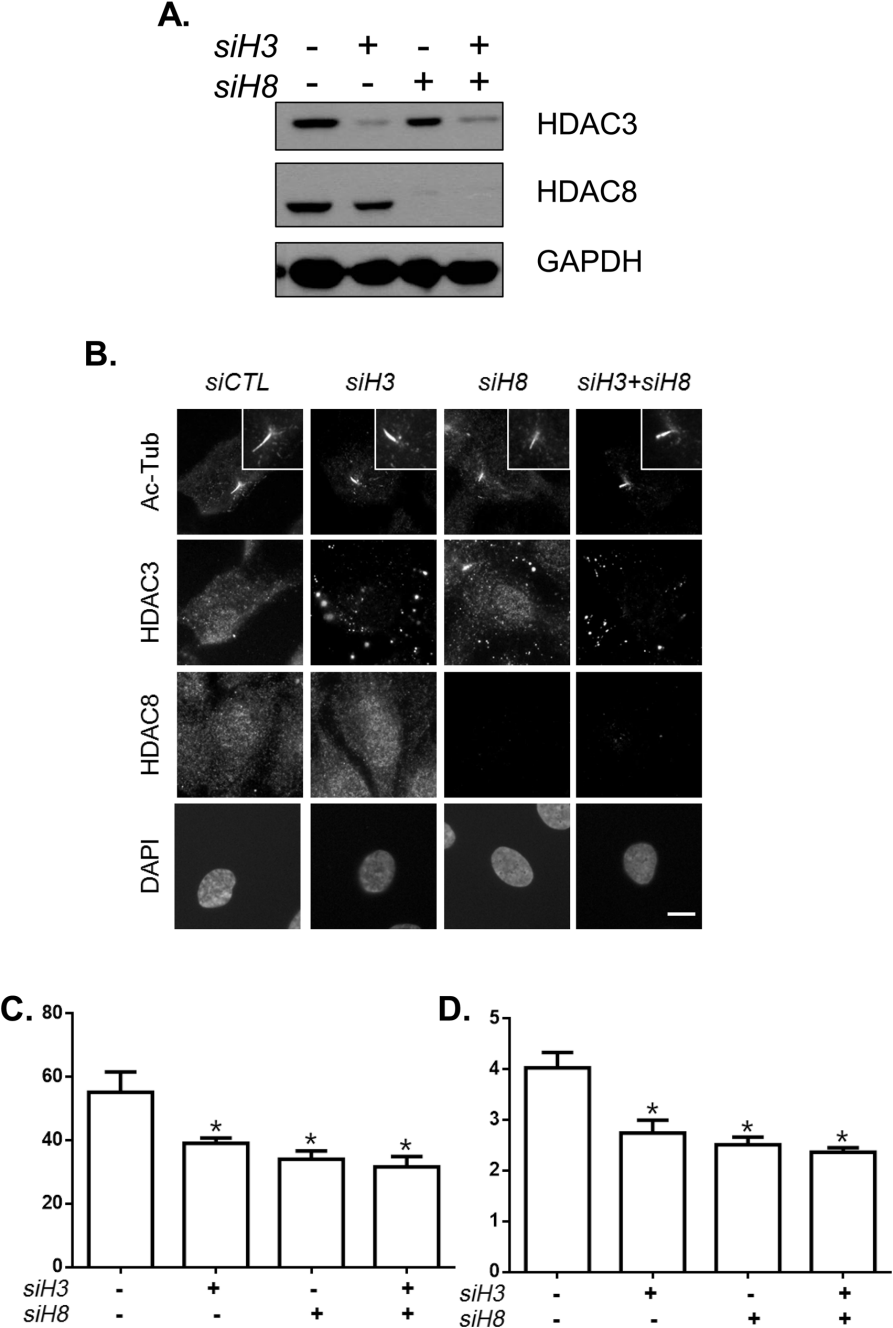
**Co-depletion of HDAC3 and HDAC8.** RPE1 cells were transfected with *siHDAC3* and/or *siHDAC8*. Twenty-four hours later, the cells were transferred to serum-deprived medium and cultured for 48 h. (A) The cells were subjected to immunoblot analysis with antibodies specific to HDAC3, HDAC8 and GAPDH. (B) The cells were co-immunostained with the antibodies specific to acetylated α-tubulin, HDAC3 and HDAC8. Scale bar: 2 μm. (C) The number of cells with cilia was determined. Greater than 100 cells per group were counted in three independent experiments. (D) Ciliary length was measured. Greater than 30 cilia per experimental group were measured in three independent experiments. Values are means and s.e.m. Statistical significance was analyzed using two-way ANOVA and is indicated by *(*P*<0.05).

To determine how HDACs are involved in cilia assembly processes, we depleted both HDAC3 and HDAC8 and determined the number of cells with cilia and ciliary lengths. Immunoblot and immunostaining analyses revealed that both HDAC3 and HDAC8 were depleted in RPE1 cells (Fig. 5A,B). As expected, the number of cells with cilia and ciliary lengths were reduced in the individually depleted cells compared to those in control RPE1 cells (Fig. 5B–D). The depletion of HDAC3 and HDAC8 also reduced the number of cells with cilia and ciliary lengths, but their effects were no more than those following their individual depletion (Fig. 5B–D). These results suggest that HDAC3 and HDAC8 participate in the same pathway during the cilia assembly process.

### HDAC3 and HDAC8 are not involved in cilia disassembly

**Fig. 6.**
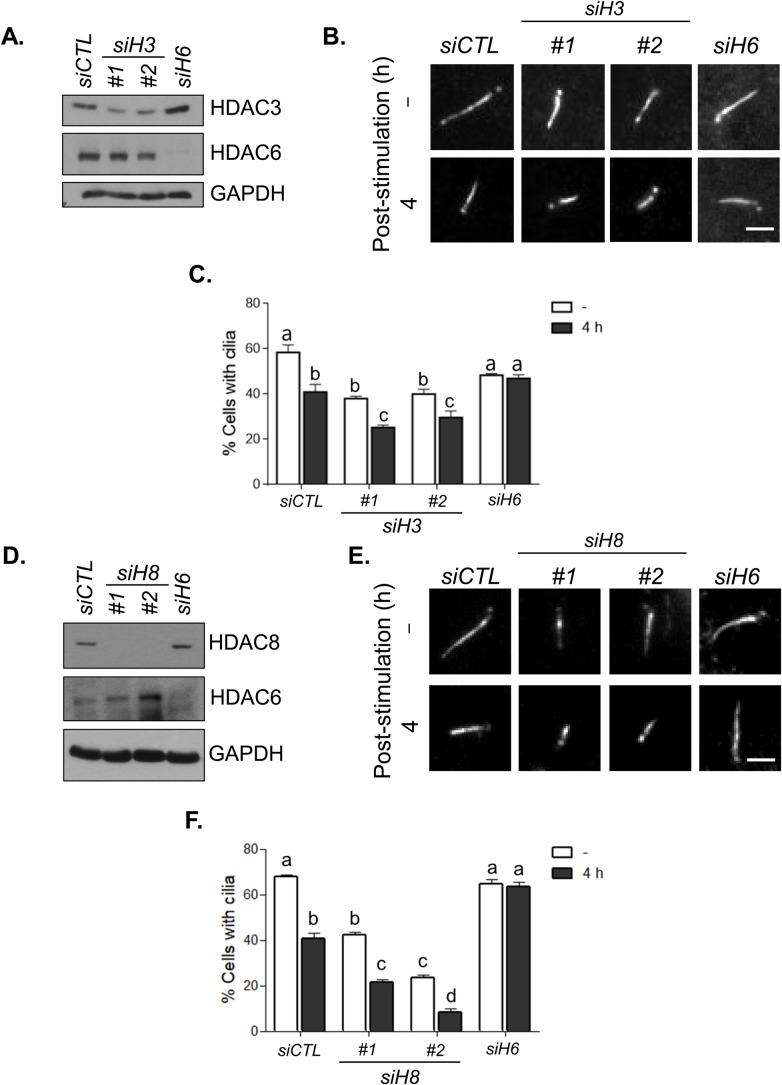
**HDAC3 and HDAC8 are not involved in the cilia disassembly.** HDAD3- (A–C) and HDAD8-depleted (D–F) RPE1 cells were cultured in serum-deprived medium for 48 h and then transferred to serum-containing medium for 4 h. HDAC6-depleted cells were included as a control. (A,D) The cells were subjected to immunoblot analysis to confirm knockdown efficiency. (B,E) The cells were immunostained with the acetylated α-tubulin antibody. Scale bars: 2 μm. (C,F) The number of cells with cilia was determined. Greater than 100 cells per group were counted in three independent experiments. Values are means and s.e.m. Statistical significance was analyzed using two-way ANOVA and is indicated by lower case letters (*P*<0.05).

HDAC6 is known to deacetylate α-tubulin in the microtubules of the axoneme and is therefore essential for cilia disassembly (Plotnikova et al., 2012). We determined whether HDAC3 and HDAC8 are involved in cilia disassembly. RPE1 cells were depleted of HDAC3 and HDAC6 and cultured in serum-deprived medium for 48 h to induce cilia assembly (Fig. 6A,B). Consistent with the previous data (Fig. 4), cilia formation was significantly reduced in HDAC3-depleted cells but not in HDAC6-depleted cells compared to cilia formation in control cells (Fig. 6B,C). Disassembly of the cilia was initiated 4 h after transferring the cells into serum-containing medium. The number of cells with cilia was rapidly reduced in the control and HDAC3-depleted cells but not in HDAC6-depleted cells (Fig. 6C). We also observed identical results in HDAC8-depleted RPE1 cells (Fig. 6D–F). These results suggest that, unlike HDAC6, HDAC3 and HDAC8 are not implicated in cilia disassembly.

## DISCUSSION

In this work, we identified HDACs that are required for the assembly and elongation of the cilia. The depletion of HDAC3 and HDAC8 significantly reduced the number of cells with cilia in RPE1 and HK2 cells. The ciliary length was also reduced. This is the first report that specific HDACs are required for cilia assembly and elongation. The biological functions of HDAC3 and HDAC8 are different than those of HDAC6, which plays a role in cilia disassembly (Pugacheva et al., 2007).

When cells were cultured in serum-deprived medium, the number of assembled cilia continuously increased in parallel with the number of cells entering G0/G1 phase (Sanchez and Dynlacht, 2016). However, in our experimental conditions, most of the HDAC3- and HDAC8-depleted cells had already entered G0/G1 phase (Fig. S2). Therefore, we ruled out the possibility that the depletion of HDAC3 and HDAC8 indirectly inhibits cilia assembly by regulating cell cycle progression. Rather, our knockdown experiments revealed a positive correlation between the cilia assembly and cellular levels of HDAC8, suggesting that HDAC8 and possibly HDAC3 directly participate in cilia assembly.

Our co-depletion experiments suggested that HDAC3 and HDAC8 play roles in the same pathway during the cilia assembly process (Fig. 5). At which step HDAC3 and HDAC8 function in cilia assembly remains to be investigated. A clue may reside in a previous report in which giantin, a Golgi matrix protein, was shown to be required for ciliogenesis (Asante et al., 2013). The depletion of giantin induced a reduction in cilia formation by controlling the localization of dynein-2 to the pericentriolar material (Asante et al., 2013). It is interesting that the localization of giantin in the juxtanuclear region is regulated by HDAC8 (Yamauchi et al., 2011). In fact, the depletion of HDAC8 caused the dispersal of giantin throughout the cytoplasm (Yamauchi et al., 2011). Therefore, one possible scenario may be that HDAC8 induces cilia assembly by controlling the juxtanuclear localization of giantin. It was also reported that HDAC8 was required to maintain Notch1 protein stability by protecting against degradation mediated by Fbxw7 in a deacetylase activity-independent manner in breast cancer (Chao et al., 2016). Since Notch signaling regulates cilia elongation, another possible scenario may be that HDAC8 controls ciliary length by regulating the ubiquitin-mediated degradation of Notch1 (Lopes et al., 2010; Tözser et al., 2015). However, we did not rule out the possibility that HDAC3 and HDAC8 may indirectly induce ciliogenesis by controlling expression of selected genes for cilia assembly and elongation.

It was recently revealed that HDAC inhibitors suppress polycystic kidney disease (PKD) and are associated with the structure and function of abnormal cilia (Huang and Lipschutz, 2014). Treatment with TSA or valproic acid, inhibitors of class I HDACs, affected body curvature, a straightforward surrogate marker for the cystic kidney phenotype, and laterality in zebrafish embryos but not adult zebrafish (Cao et al., 2009). However, it also suppressed the body curvature phenotype in zebrafish *pkd2* mutants. These results were further validated by mouse studies (Cao et al., 2009; Xia et al., 2010). Therefore, HDAC inhibitors may be applied for PKD treatment and further ciliopathy treatment. In fact, selected HDAC inhibitors have been applied in several diseases, such as mood disorder, epilepsy and cancer. We are currently looking for specific targets of HDAC3 or HDAC8 in cilia assembly and elongation.

## MATERIALS AND METHODS

### Cell culture and drug treatment

hTERT-RPE1 and HK2 cells were cultured in DMEM/F-12 (WelGENE, LM 002-04) supplemented with 10% fetal bovine serum (FBS) (WelGENE, S101-01) and Plasmocin™ (InvivoGen, anti-mpt). To induce primary cilia assembly, the amount of FBS in the medium was reduced to 0.1%. TSA (Sigma-Aldrich, T8552) was added to RPE1 cells for 12 h to inhibit HDAC activity.

### Transfection of siRNAs and plasmids

ST Pharm synthesized siRNAs specific to *HDAC1* (*siHDAC1*; 5′ - GCU UCA AUC UAA CUA UCA ATT - 3′), *HDAC2* (*siHDAC2*;  5′ - CCC AAU GAG UUG CCA UAU AAU TT - 3′), *HDAC3* (*siHDAC3 #1*; 5′ - CAA CAA GAU CUG UGA UAU UTT - 3′ and *siHDAC3 #2*; 5′ - GCA CCC GCA UCG AGA AUC ATT - 3′), and *HDAC8* (*siHDAC8*; 5′ - GCU GGG AGC UGA CAC AAU ATT - 3′). siRNAs specific to *HDAC1* (L-003493-00-0005), *HDAC2* (L-003495-02-0005), *HDAC8* (M-003500-02-0005) and *HDAC6* (J-003499-05-0005) were purchased from Dharmacon. The control siRNA was scrambled (*siCTL*; 5′- GCA AUC GAA GCU CGG CUA CTT - 3′). The siRNAs were transfected into RPE1 and HK2 cells with RNAiMAX (Invitrogen, 13778-075).

The siRNA recognition site of *HDAC3* was silently mutated using the 5′- CAT CCA GAT GTC AGC ACC CGC ATC GAG AAT CAG AAC TCA CGC CAG - 3′ primer. The deacetylase-dead HDAC3 mutant includes an arginine substitution to proline at residue 265 (Watson et al., 2012). The HDAC3 constructs were subcloned into the *pLVX-IRES-Puro-3xFlag* vector. RPE1 cells were stably transfected with the HDAC3-expresssing vectors.

The siRNA recognition site in *HDAC8* was silently mutated using the 5′- GCT AGG TGC CGA TAC CAT T -3′ primer. The deacetylase-dead HDAC8 mutant included a histidine substitution to alanine at residues 142 and 143 (Gantt et al., 2016). The HDAC8 constructs were subcloned into the *pLVX-IRES-Puro-3×Flag* vector. RPE1 cells were stably transfected with the HDAC8 expression vectors.

### Antibodies

The primary antibodies used were specific to HDAC1 (Abcam, ab31263), HDAC2 (Abcam, ab12169), HDAC3 (Abcam, ab7030), HDAC8 (Proteintech, 17548-1-AP), HDAC6 (Abgent, AP1106a), acetylated α-tubulin (Cell Signaling Technology; 5335S, Sigma-Aldrich; T6793), ARL13B (Proteintech, 17711-1-AP), Flag (Sigma-Aldrich, F3165), or GAPDH (Ambion, AM4300). The secondary antibodies used were conjugated to fluorescent dyes (Alexa-488, Alexa-555, Alexa-594, and Alexa-647; Life Technologies) or horseradish peroxidase (mouse, Sigma-Aldrich; rabbit, Millipore).

### Immunoblot analysis

Specific proteins were detected as previously described (Seo et al., 2015). In brief, the cells were first washed with PBS and lysed with lysis buffer (50 mM Tris, pH 7.5, 150 mM NaCl, 0.5% NP-40, 0.5% Triton X-100, 20 mM NaF, 20 mM β–glycerophosphate, 1 mM Na_3_VO_4_) containing protease inhibitor cocktail (Sigma-Aldrich) for 30 min on ice. After centrifugation, the soluble fraction was used for protein quantification by Bradford assay. For each tested group, 15–25 µg of proteins were boiled with Laemmli sample buffer and subjected to SDS-PAGE. After the proteins were transferred onto a nitrocellulose membrane, it was blocked in 5% skimmed milk in TBST (20 mM Tris-Cl, pH 8.0, 150 mM NaCl and 0.1% Tween 20) for 1 h and incubated with primary antibodies overnight at 4°C. The membrane was then washed three times with TBST. After incubation with HRP-conjugated secondary antibodies for 1 h, the membrane was washed three times with TBST and visualized with ECL solutions.

### Immunofluorescence microscopy and ciliary length measurements

Cells were subjected to immunostaining as previously described (Kim et al., 2015a). In brief, cells grown on 12-mm coverslips were fixed with cold methanol on ice for 10 min and permeabilized with PBST (0.3% Triton X-100 in PBS) for 10 min. Cells were blocked with blocking buffer (3% bovine serum albumin and 0.3% Triton X-100 in PBS) for 20 min, incubated with the indicated primary antibody for 1 h, washed three times with PBST, incubated with the indicated secondary antibody for 30 min, washed three additional times with PBST, counterstained with a DAPI solution for 1 min, and mounted onto glass slides with ProLong Gold antifade reagent (Invitrogen, P36930). Cells were observed under a fluorescence microscope (Olympus IX51) with a 60×/1.25 oil iris (UFlanFl) objective lens. Images were acquired on a CCD camera (QICAM Fast 1394, QImaging) and analyzed using ImagePro 5.0 (Media Cybernetics, Inc.). Ciliary lengths were measured using the NeuronJ plugin with ImageJ software (National Institutes of Health).

### Statistical analysis

All experiments were repeated at least three times. GraphPad Prism 5 was used for one-way ANOVA, and SigmaPlot 12.0 (Systat Software) was used for two-way ANOVA.

## Supplementary Material

Supplementary information
