# Supplementary material for: HDAC3 and HDAC8 are required for cilia assembly and elongation
Source: Biol Open. 2019 Jul 30;8(8):bio043828. doi: 10.1242/bio.043828 (PMC6737963; doi:10.1242/bio.043828)
Supplement: Supplementary information [file biolopen-8-043828-s1.pdf]

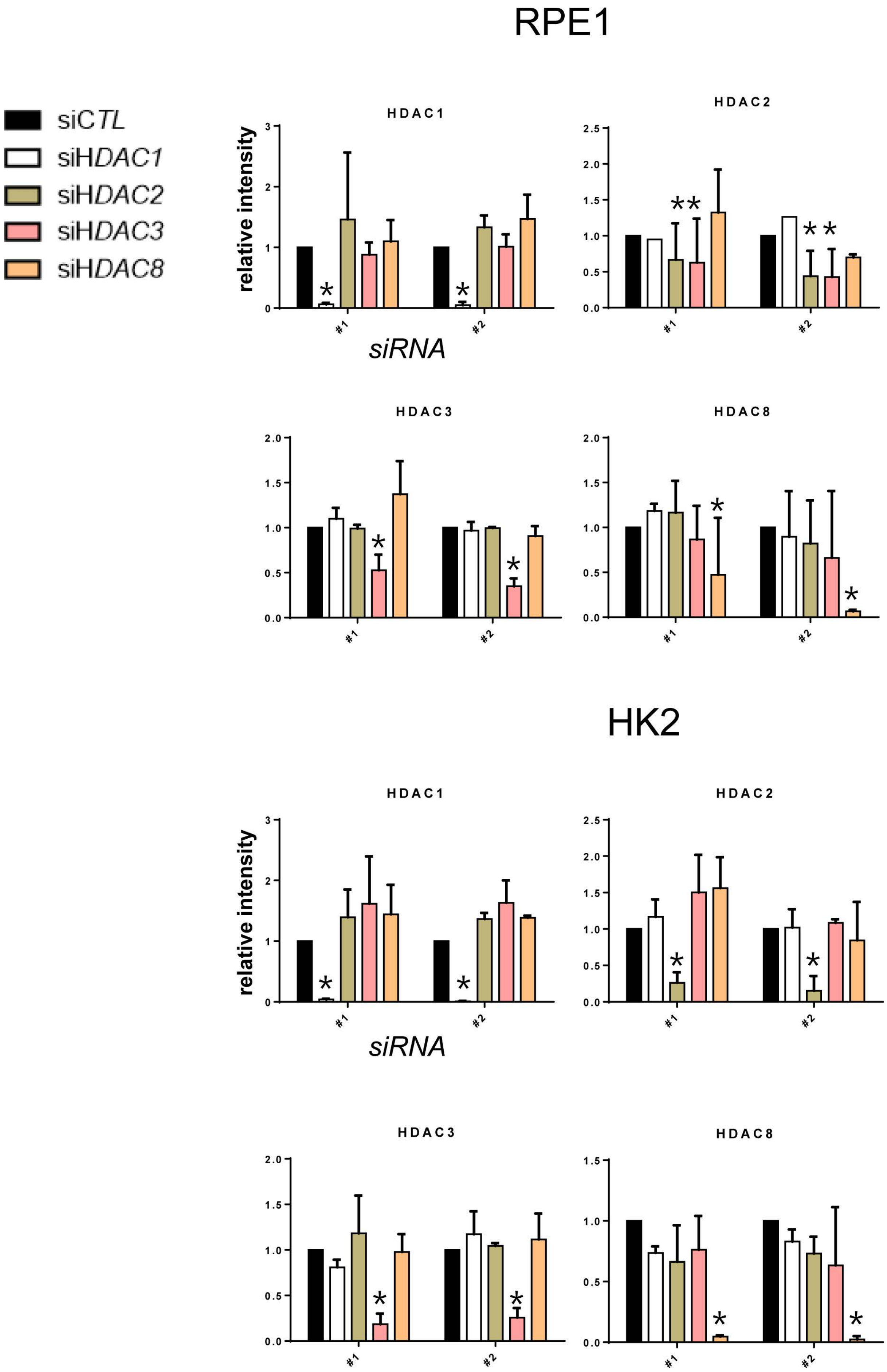

Biology Open • Supplementary information

Figure S1. Cellular HDAC levels in siRNA-transfected cells. Band intensities in the Figure 2A experiment was measured and normalized with the GAPDH band intensities. The experiments were repeated 3 times. Values are means and SEM. Statistical significance was analysed using two-way ANOVA and is indicated by * ($P<0.05$).

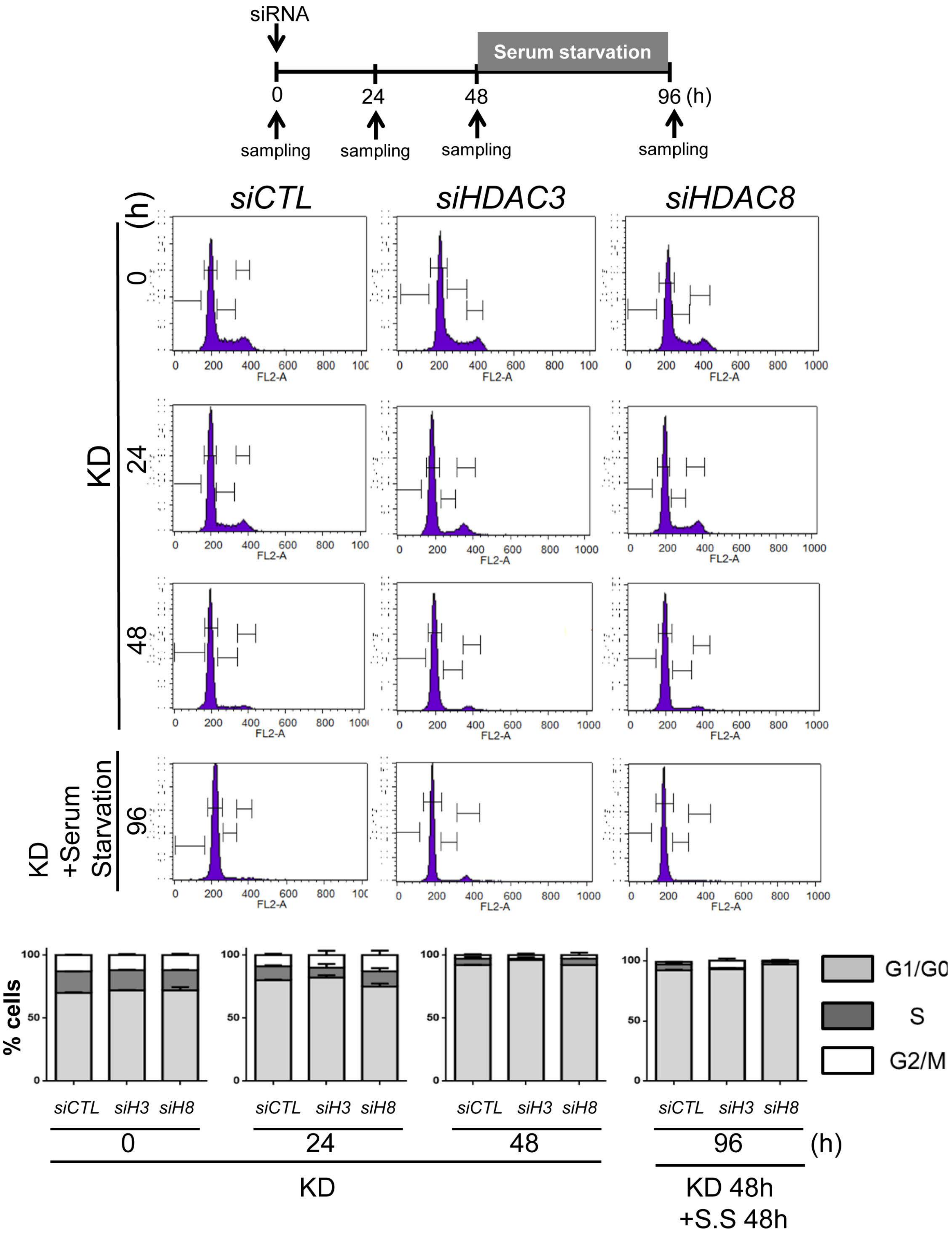

Biology Open • Supplementary information

Figure S2. FACS analysis of the HDAC-depleted cells cultured in serum-deprived medium. RPE1 cells were transfected with siRNAs specific to HDAC3 and HDAC8 and cultured for 48 h. The cells were then transferred in serum-deprived medium and cultured for additional 48 h. The cells at indicated stages were subjected to FACS analysis to determine cell cycle stages at indicated time points. The experiments were repeated 3 times. Values are means and SEM.

Biology Open • Supplementary information

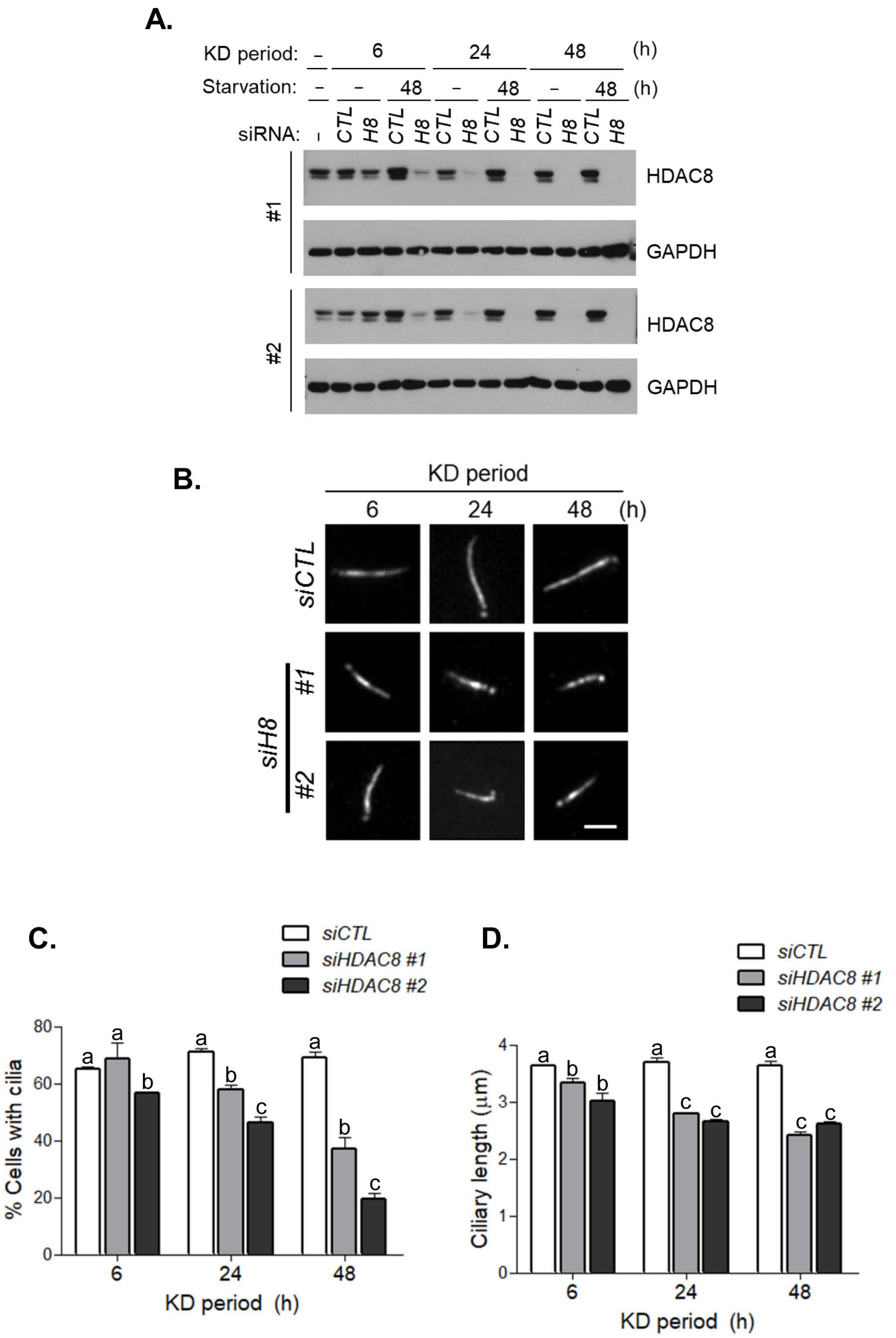

Biology Open • Supplementary information

Figure S3. HDAC8 is required for the formation and elongation of cilia. RPE1 cells were transfected with *siHDAC8* and cultured for 6, 24, or 48 h. The cells were then transferred to serum-deprived medium for 48 h. (A) The cells were subjected to immunoblot analysis with antibodies specific to HDAC8 and GAPDH. (B) The cells were immunostained with the acetylated α-tubulin antibody. Scale bar, 2 µm. (C) The number of cells with cilia was determined. Greater than 100 cells per group were counted in 3 independent experiments. (D) Ciliary length was measured. Greater than 30 cilia per experimental group were measured in 3 independent experiments. Values are means and SEM. Statistical significance was analysed using one-way ANOVA and is indicated by lower cases ($P<0.05$).

Table S1. List of classes I, II and IV HDACs. HDACs are grouped into classes I, II and IV, based on their structural similarity as described in Yang and Seto (2008).

Class I	HDAC1, HDAC2<br>HDAC3, HDAC8	
Class II	Class IIa	HDAC4<br>HDAC5<br>HDAC7<br>HDAC9
	Class IIb	HDAC6<br>HDAC10
Class IV	HDAC11	
